# *TWIST1* Is Expressed in Colorectal Carcinomas and Predicts Patient Survival

**DOI:** 10.1371/journal.pone.0018023

**Published:** 2011-03-28

**Authors:** Irene Gomez, Cristina Peña, Mercedes Herrera, Concepción Muñoz, Maria Jesus Larriba, Vanesa Garcia, Gemma Dominguez, Javier Silva, Rufo Rodriguez, Antonio Garcia de Herreros, Felix Bonilla, Jose M. Garcia

**Affiliations:** 1 Department of Medical Oncology, Hospital Universitario Puerta de Hierro de Majadahonda, Majadahonda, Madrid, Spain; 2 Unitat de Biología Cellular i Molecular, Institut Municipal d'Investigació Mèdica, Universitat Pompeu Fabra, Barcelona, Spain; 3 Department of Gastroenterology, Hospital Virgen de la Salud, Toledo, Spain; 4 Department of Pathology, Hospital Virgen de la Salud, Toledo, Spain; 5 Instituto de Investigaciones Biomédicas “Alberto Sols”, Consejo Superior de Investigaciones Científicas-Universidad Autónoma de Madrid, Madrid, Spain; Florida International University, United States of America

## Abstract

TWIST1 is a transcription factor that belongs to the family of basic helix-loop-helix proteins involved in epithelial-to-mesenchymal transition and invasion processes. The TWIST1 protein possesses oncogenic, drug-resistant, angiogenic and invasive properties, and has been related with several human tumors and other pathologies. Colorectal cancer is one of the tumors in which TWIST1 is over-expressed, but its involvement in the clinical outcome of the disease is still unclear. We tested, by RT-PCR, the expression levels of *TWIST1* in normal and tumor paired-sample tissues from a series of 151 colorectal cancer patients, in order to investigate its prognostic value as a tumor marker. *TWIST1* expression was restricted to tumor tissues (86.1%) and correlated with lymph node metastasis (LNM). Adjusted analysis showed that the expression levels of *TWIST1* correlated with overall survival (OS) and disease-free survival (DFS). Importantly, *TWIST1* expression levels predicted OS specifically at stages I and II. Moreover, patients with stage II tumors and high *TWIST1* levels showed even shorter survival than patients with stage III tumors. These results suggest that *TWIST1* expression levels could be a tumor indicator in stage II patients and help select patients at greater risk of poor prognosis who might benefit from adjuvant chemotherapy.

## Introduction

Colorectal cancer (CRC) is the second most common cause of cancer mortality in the developed countries and remains associated with a high mortality rate [1]. Metastases are the end result of tumor progression and the most common cause of death in cancer patients. The genetic bases for metastasis are beginning to be outlined [2].

Altered functions of several genes that are key players in embryonic development are related to some steps in oncogenesis, such as epithelial-mesenchymal transition (EMT). During gastrulation, certain cells from an epithelial-like structure undergo EMT and migrate to organize the mesoderm embryonic layer. The theory that tumor cells trigger EMT to allow migration and invasion has received considerable attention, since several genes involved in EMT during embryogenesis are turned up during oncogenesis [3]. Yang and co-workers, in a mouse model of breast cancer, identified genes related to each step of metastasis, particularly those involved in invasion and intravasation steps in which EMT is a necessary process [4]. In this context, the transcription factor TWIST1 was identified as an essential protein in the intravasation step. TWIST1 induces EMT in epithelial cells by activation of SNAI2 transcription [5], repression of E-cadherin-mediated cell-cell adhesion and acquisition of mesenchymal markers such as fibronectin and N-cadherin. Moreover, significant correlation was found between the expression of TWIST1 and the appearance of invasive lobular carcinomas [4]. This corroborated a previous study in which the *TWIST1* promoter was much less frequently methylated in invasive lobular carcinomas than in invasive ductal carcinomas [6].

A number of studies indicate that TWIST1 possesses oncogenic [7]–[10], drug-resistant [11]–[12], angiogenic [13] and invasive [11], [14]–[17] properties. In addition, TWIST1 over-expression has been found in tumor tissues such as rhabdomyosarcoma [7], melanoma [8], pediatric osteosarcoma [18], T-cell lymphoma [10], gastric [19], prostate [11] and breast carcinoma [4], [17], [20]. Recently, it has been demonstrated that the TWIST1 protein overrides oncogene-induced senescence both in murine and human cancer cells [21].

*TWIST1* over-expression in colorectal cancer is associated with gender and with a poor prognosis factor, such as nodal invasion [22], but its impact on the clinical outcome of the disease is still not clear. At the moment, pathological staging is still the most useful prognostic factor [23]. However, new molecular or clinical parameters are needed, to improve the current methods for deciding which patients could benefit from adjuvant chemotherapy and when.

We hypothesize that the expression level of *TWIST1* in primary colorectal tumors determines the characteristics of the tumors, their behavior and their clinical outcome.

## Materials and Methods

### Patients and samples

The present study was based on a consecutive series of 151 patients undergoing surgery for colorectal cancer and included in a prospective study. Informed written consent was obtained from all participants after an explanation of the nature of the study, as approved by the Research Ethics Board of Puerta de Hierro Majadahonda University Hospital. All patients were considered sporadic cases, inasmuch as those with familial adenomatous polyposis and clinical criteria for hereditary non-polyposis colorectal cancer (Amsterdam criteria) were excluded.

Tumor and normal colon mucosa (taken at least 3 cm from the outer tumor margin) were obtained immediately after surgery, immersed in RNA*later*™ (Ambion Inc, Austin, Texas), snap-frozen in liquid nitrogen and stored at −80°C until processing. All patients in the study gave written informed consent.

### RNA extraction

RNA was extracted from tumor and normal samples with the RNeasy Mini Kit (Qiagen Inc., Hilden, Germany), according to the manufacturer's protocol. The RNA extracted was quantified with a NanoDrop ND-1000 Spectrophotometer (nanoDrop Technologies Inc., Wilmington, Delaware, USA).

### Real-Time PCR

*TWIST* mRNA expression in each sample was measured as a ratio against the geometric average of three reference housekeeping genes, *succinate dehydrogenase complex subunit A* (*SDHA*), *TATA binding protein* (*TBP*) and *ubiquitin C* (*UBC*) [24]. The relative concentrations of the target and the reference genes were calculated by interpolation, using a standard curve of each gene plotted from the same serial dilution of cDNA from tumor tissue. The quantitative mRNA analysis was performed in duplicate. *TWIST1* expression was only determined in tumor tissues, since normal tissues showed no expression of this gene. An arbitrary value (0.01), corresponding to half the minimum value detected in the series, was assigned to the tumors in which *TWIST* expression was not detected. *SNAI2* expression was calculated as the ratio of its expression in tumor (T) *vs* its expression in normal tissue (N).

The primers used were: *SDHA*-5′TGGGAACAAGAGGGCATCTG 3′ forward (F) and 5′CCACCACTGCATCAAATTCATG 3′ reverse (R); *TBP*-5′TCTGGGATTGTACCGCAGC3′ forward (F) and 5′CGAAGTGCAATGGTCTTTAGG3′ reverse (R); UBC 5′ATTTGGGTCGCGGTTCTTG3′ forward (F) and 5′TGCCTTGACATTCTCGATGGT3′ reverse (R); *TWIST1* 5′ CATGTCCGCGTCCCACTAG 3′ forward (F) and 5′ TGTCCATTTTCTCCTTCTCTGG 3′ reverse (R); *SNAI2* 5′-GGCAAGGCGTTTTCCAGAC-3′ forward (F) and 5′-GCTCTGTTGCAGTGAGGGC-3′ reverse (R). The annealing temperature in all cases was 59°C. At the end of the PCR cycles, melting curve analyses were performed to confirm the generation of the specific expected PCR product. The PCR products were sequenced in an ABI Prism™ 377 DNA sequencer apparatus (PE Applied Biosystems). For the synthesis of cDNA, 400 ng of total RNA was retro-transcribed, using the Gold RNA PCR Core Kit (PE Biosystems, Foster City, CA). Real-time PCR was performed in a Light-Cycler apparatus (Roche Diagnostics, Mannheim, Germany), using the LightCycler-FastStart^PLUS^ DNA Master SYBR Green I Kit (Roche Diagnostics, Mannheim, Germany).

### Clinico-pathological parameters of the patients

The parameters obtained from the medical records of the 151 patients were: age, tumor location, lymph node metastases (LNM) (evaluated by optical microscopy), pathological stage (assessed by the tumor-node-metastases classification), tumor histological grade and the presence of vascular invasion in tumors.

### Patients' treatment and follow-up

Colon cancer patients did not receive neo-adjuvant chemotherapy (CT). Patients with rectal carcinoma who had received preoperative treatment with CT and radiotherapy or radiotherapy alone were excluded, because of the difficulty of finding a suitable tumor for determining gene expression in these patients' surgical samples. Adjuvant treatment based on oxaliplatin (FOLFOX6, leucovorin 400 mg/m^2^ IV on day 1 as a 2-hour infusion, followed by 5-fluorouracil bolus of 400 mg/m^2^ IV on day 1, followed by 2,400 mg/m^2^ IV 46-hour infusion and oxaliplatin 100 mg/m^2^ IV as a 2-hour infusion on day 1) was administered to the 51 stage-III patients (29 colon cancer and 22 rectal cancer) without medical contra-indications who gave their written informed consent. Radiotherapy was also administered to the 22 rectal tumor cases. The median age of this subgroup of patients was 69.3 years.

Clinical follow-up after surgery and diagnosis was based on periodic visits and clinical, biochemical and imaging techniques. Ultrasonic study was performed when liver function was impaired. Overall and Disease-Free Survival (OS and DFS) were defined as the period of time from diagnosis to death and the interval between diagnosis and first recurrence, respectively.

### Statistical analysis

As the distribution of the gene expression values was not normally distributed (Kolmogorov-Smirnov test), we normalized the data distribution by using log_10_ to carry out the statistical analysis.

The clinico-pathological parameters were contrasted with *TWIST* expression data in tumor tissues by the one-way ANOVA test. The General Linear Model was applied to age and stage in order to test the possible interaction between the two variables, as well as their independent value in relation to *TWIST1 mRNA* expression levels.

To study OS and DFS, the expression data of *TWIST* were divided by tertiles. The expression levels defining the three groups for the *TWIST* gene were 0.56 (33%) and 1.7 (66%). DFS analysis did not include patients at pathological stage IV. The relationship between the cumulative probability of OS and DFS, as well as analyzed predictors, was calculated with the Kaplan-Meier method [25], while significant differences between curves were evaluated with Mantel's log-rank test [26]. To identify factors that might be of independent significance in influencing OS and DFS, multivariate analysis (Cox proportional risk regression model) was applied [27]. Confounding and interacting variables were analyzed. The model's basic assumptions (proportional hazards) were evaluated. In all statistical tests two-tailed *p* values ≤ 0.05 were considered statistically significant. Statistical analyses were performed with SPSS 13.0 statistical software (SPSS Inc, Chicago, IL).

## Results

### *TWIST1* expression is confined to tumor tissues in human colorectal cancer and is up-regulated in patients with lymph node metastasis

The analysis of *TWIST1* expression levels in tumor and normal matched tissues from 151 patients with colorectal cancer showed that the expression of *TWIST1* is restricted to the tumor mass. It was never detected in any normal tissues. Of 151 tumor samples tested, *TWIST1* was detected in 130 cases (86.1%).

A statistical association between high levels of *TWIST1* in tumor tissues and lymph node metastasis (LNM) was observed (*p* = 0.016), while only a trend to statistical association was found for age (*p* = 0.052) and tumor stage (*p* = 0.07) (Table 1). Though this suggests a possible interaction between the two variables, the statistical General Linear Model showed no interaction between age and tumor stage, as well as, an independent relationship for age and *TWIST1* expression levels, *p* = 0.031. The median and range of lymph node harvesting counts were: 9 (0–34).

**Table 1:** Associations between the expression of *TWIST* gene in tumor tissues and clinico-pathological characteristics.

Characteristics	Total (%)	Expression of *TWIST*	
		Median/minimum/maximum	*p* a
Patients	151		
Median age	71		
<71	63 (41.72%)	0.92/0.001/10.33	0.052
>71	88 (58.28%)	1.04/0.01/56.90	
Gender			
Male	96 (63.58%)	1.04/0.01/56.90	NS
Female	55 (36.42%)	0.88/0.01/23.63	
Tumor side			
Colon	102 (67.55%)	0.91/0.01/56.90	NS
Rectum	49 (32.45%)	1.67/0.01/22.78	
Stage			
I	14 (9.27%)	1.53/0.01/3.89	0.074
II	75 (49.67%)	0.89/0.01/56.90	
III	51 (33.77%)	1.16/0.01/22.78	
IV	11 (7.28%)	1.67/0.44/23.66	
Vascular invasion			
Yes	63 (41.72%)	0.96/0.01/56.90	NS
No	88 (58.28%)	1.00/0.01/23.63	
Lymph node metastases			
Positive	62 (41.06%)	1.19/0.01/23.63	0.016
Negative	89 (58.94%)	0.94/0.01/56.90	
Tumor differentiation			
Good	41 (27.15%)	0.82/0.01/7.04	NS
Moderate	77 (50.99%)	1.22/0.01/22.78	
Poor	33 (21.85%)	1.67/0.01/56.90	

a *p* is calculated by the ANOVA test.

NS: Not significant.

No statistical associations between *TWIST1* expression levels and the other pathological variables analyzed were found (summarized in Table 1).

### *TWIST1* mRNA levels correlate with the transcriptional activity of the protein

**Figure 1:**
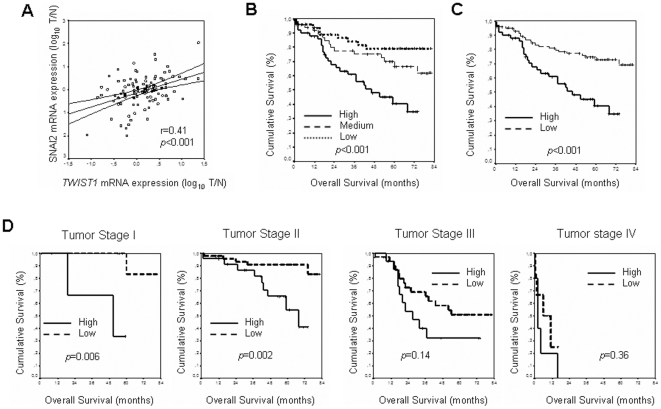
Kaplan-Maier OS curves and TWIST1 activity. A, Relation between expression levels of *TWIST1* and *SNAI2* genes, logR(T/N), in patients in which *TWIST1* was previously detected. *TWIST1* and *SNAI2* expression directly correlates in human colon cancer, Pearson correlation coefficient *r* = 0.41 (*P*<0.001). Kaplan-Meier curves and *p* values for OS for *TWIST1* expression levels: B, in the series distributed by tertiles: low, medium and high. C, grouping tertiles: low and medium as low. D, stratifying the series by tumor stage.

To ensure that *TWIST1* mRNA levels detected in each sample correlate with protein activity, the mRNA levels of Snai2 were measured in the 130 patients in whom *TWIST1* was previously detected. SNAI2 is another transcription factor involved in the EMT, whose transcription is directly regulated by TWIST1, as has been recently demonstrated [5]. As expected, a direct correlation between *TWIST1* and *SNAI2* mRNA levels (Pearson correlation coefficient *r* = 0.41, *P*<0.001) was found, suggesting that there is a correlation between *TWIST1* mRNA levels and protein activity (Fig 1A).

### *TWIST1* expression level is related to overall survival

The series was followed for a mean of five years (range of patient follow-up: 1–82 months). During this period, 54 recurrences (35.8%) were recorded and 50 patients (33.1%) died, with the five-year OS for the series at 62.4% (95% CI, 53.8%–70.98%). To carry out survival analysis, the series was divided by tertiles on the basis of *TWIST1* expression levels. Thus, patients were classified with low, medium or high levels of *TWIST1* expression. A statistical difference was observed in OS for the expression of *TWIST1* (*p*<0.001): the five-year OS for each group was 79% (95% CI, 67%–91%) for those patients with low expression levels; 66.6% (95% CI, 51.9%–81.3%) for patients with medium expression levels; and 40.6% (95% CI, 24.6%–56.6%) for patients with high expression levels (Fig. 1B). Since no statistical differences were found for OS in patients with low or medium levels of *TWIST1* expression and both groups behaved quite similarly (see Fig. 1B), unlike patients with high *TWIST1* expression levels, these two categories were grouped. Therefore, further studies were carried out with only two categories: patients with low (the former low plus medium levels) or high expression levels of *TWIST1*. No changes in the correlation previously observed between OS and *TWIST1* expression levels were found with this new classification. Thus, five-year OS for patients with high expression levels was the above-mentioned 40.6% (95% CI, 24.6%–56.6%), versus 72.9% (95% CI, 63.3%–82.5%) in those cases with low expression levels (*p*<0.001) (Fig. 1C).

Since, according to this result, the expression levels of *TWIST1* in human colorectal cancer could be considered a poor prognosis factor, we were interested in the clarification of its possible prognosis value at each different colorectal tumor stage. The number of patients with low expression levels was: 9 for stage I, 51 for stage II, 34 for stage III and 6 for stage IV. Equally, the number of patients with high expression levels was: 5 for stage I, 24 for stage II, 17 for stage III and 5 for stage IV. Interestingly, the Kaplan-Meier analysis showed that the expression levels of *TWIST1* correlated with OS, but only in stages I and II. Therefore, five-year OS for stage I was 33.3% (95% CI, 0%–86.65%) in patients with high levels *versus* 83.3% (95% CI, 53.5–100%) in patients with low expression levels (*p* = 0.006); for stage II, it was 54.53% (95% CI, 28.29%–80.77%) in patients with high expression levels *versus* 90.92% (95% CI, 82.42–99.42%) for patients with low expression levels (*p* = 0.002). In contrast, the Kaplan-Meier curves in stages III or IV were similar for both groups, i.e. patients with high and low *TWIST1* expression levels, with no statistical differences observed (Fig. 1D).

**Figure 2:**
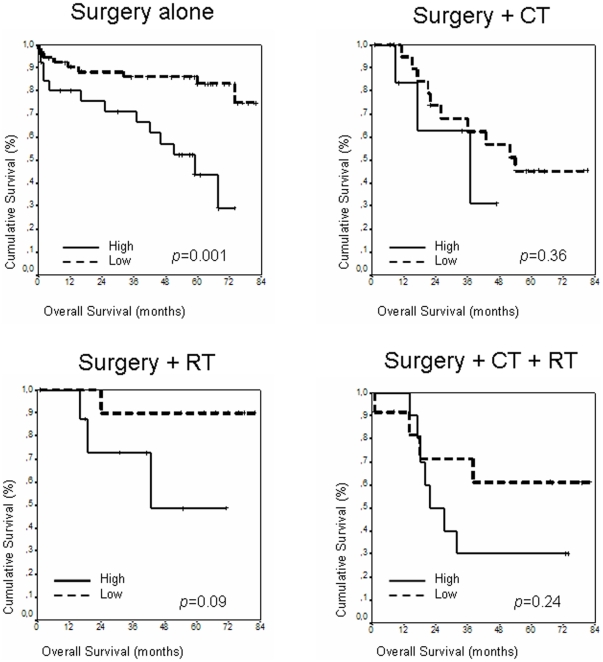
Kaplan-Maier OS curves regarding treatment protocols. Kaplan-Meier curves and *p* values for OS for *TWIST1* expression levels in each one of the treatment groups. CT, chemotherapy; RT, radiotherapy.

Because the different treatment protocols may affect OS and mimic the prognosis value of *TWIST1*, we also analyzed the prognosis value of *TWIST1* at each different treatment subgroup (Fig 2). Four groups of treatment were identified. In the first group, 67 patients underwent only surgery. All of these were colon cancer cases with tumors at stages I or II (note that there were 6 patients with stage IV colon tumors and 8 patients with stage I or IV rectal tumors who also underwent only surgery, but were not considered in this analysis in order to achieve greater homogeneity for this study). Five-year OS for patients with high *TWIST1* expression was 54.5% (95% CI; 81.5%–27.4%) *versus* 89.6% (95% CI; 99.4%–79.8%) in patients with low expression levels (*p* = 0.001). In a second group, 19 patients underwent radiotherapy as well as surgery: all of these were patients with stage II rectal tumors, except for two at stage I. In this group, five-year OS for patients with tumors with high expression levels was 48.6% (95% CI; 92.9%–4.3%) *versus* 90% (95% CI; 100%–71.6%) in patients with low expression levels (*p* = 0.09). No differences in OS according to *TWIST1* expression levels were observed in the two remaining groups. One of these consisted of 29 patients who received chemotherapy after surgery (all were patients with stage III colon cancer tumors). The other consisted of 22 patients who received chemotherapy plus radiotherapy, as well as surgery (all of these were patients with stage III rectal cancer tumors). Again, the only clear differences in OS were found in the group considered to have good prognosis.

Univariate analysis was performed to determine the influence of *TWIST* expression and the clinico-pathological parameters in OS. Variables which could be considered statistically supported factors in OS prediction were: LNM, stage, treatment protocols and *TWIST1* expression levels (Table 2). In the multivariate Cox's regression model for OS, the variables that showed an independent prognostic factor were: LNM HR 4.02 (95% CI; 2.21–7.3) (*p*<0.001) and TWIST1 expression levels HR 2.73 (95% CI; 1.54–4.84) (p = 0.001) (Table 2). Because LNM and tumor stage are linearly dependent covariates (tumor stages I and II are LNM negatives and tumor stage III and, probably, the vast majority at tumor stage IV are LNM positives), the variable tumor stage was not included in the multivariate analysis.

**Table 2:** Unadjusted and adjusted analyses of the association between *TWIST* expression and overall survival of colon cancer patients.

Variable	Category	Unadjusted analysis			Adjusted analysis		
		HR	(95% CI)	*p* Value	HR	(95% CI)	*p* Value
Age at diagnosis	<71 vs >71	0.775	0.44–1.37	0.38			
Sex of patients	Male vs female	1.53	0.82–2.86	0.18			
Lymph node metastases	Yes vs No	3.99	2.2–7.24	<0.001	4.02	2.21–7.3	<0.001
Vascular invasion	Yes vs No	1.66	0.94–2.93	0.079			
Stage	II vs I	0.91	0.26–3.18	0.87			
	III vs I	3.06	0.92–10.2	0.068			
	IV vs I	35.09	7.89–155.9	<0.001			
Histological grade	2 vs 1	1.49	0.76–2.94	0.25			
	3 vs 1	1.02	0.42–2.5	0.96			
Tumor side	Rectum vs colon	1.41	0.79–2.50	0.24			
*TWIST* expression	High vs low	2.72	1.53–4.82	0.001	2.73	1.5–4.84	0.001
Treatment Protocols	CT *vs* surg	2.18	1.08–4.39	0.029			
	RT *vs* surg	0.77	0.26–2.25	0.63			
	CT + R *vs* surg	2.19	1.04–4.58	0.038			

The blank cells correspond to variables that showed no independent relationship with OS in the adjusted analysis. CT, chemotherapy; RT, radiotherapy.

Since our results suggested that *TWIST1* expression levels have prognosis value at early stages I and II (both LNM negatives), we repeated the Cox's regression models, stratifying the series for their LNM status. This analysis confirmed that *TWIST1* expression levels have prognosis value only in patients without lymph node metastasis (Table 3).

**Table 3:** Analysis of the association between *TWIST* expression and overall survival of colon cancer patients by lymph node metastases.

Variable	Category	LNM positives			LNM negatives		
		HR	(95% CI)	*p* Value	HR	(95% CI)	*p* Value
Age at diagnosis	<71 vs >71	2.28	0.87–5.97	0.09	0.78	0.38–1.62	0.5
*TWIST* expression	High vs low	6.57	2.31–18.7	<0.001	1.84	0.88–3.84	0.1

### *TWIST1* expression levels are related to Disease-Free Survival

**Figure 3:**
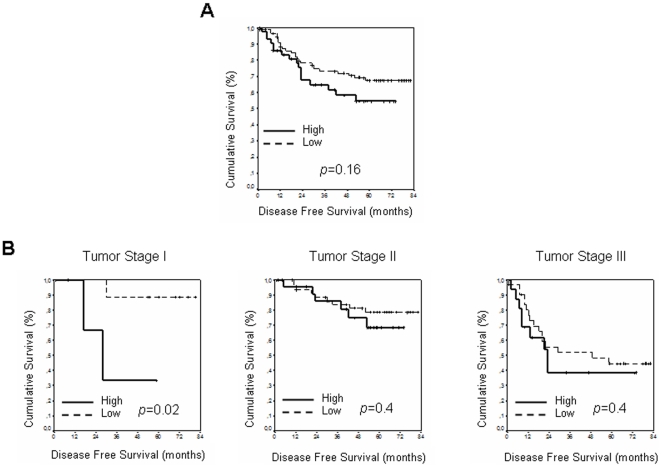
Kaplan-Maier DFS curves. Kaplan-Meier curves and *p* values for DFS for *TWIST1* expression levels: low (formed by medium and low tertiles) and high: A, in the entire series, except patients with stage IV tumor; B, at each tumor stage except stage IV.

No clear difference was observed for *TWIST1* expression levels and DFS after Kaplan-Meier analysis: five-year DFS for patients with low *TWIST1* expression levels was 67.58% (95% CI; 57.25%–77.9%) vs 54.75% (95% CI; 39.07%–70.43%) in patients with high expression levels (Fig. 3A). However, this analysis performed in the series stratified by stage showed a correlation between *TWIST1* expression levels and DFS in stage I: 88.9% (95% CI; 68.36%–100%) in patients with low expression levels vs 33.3% (95% CI; 0%–86.6%) in patients with high expression levels (*p* = 0.02). No correlation was found in stages II and III (Fig. 3B).

Variables which could be considered statistically supported factors in DFS prediction, according to the Cox's model, were LNM (*p*<0.001) and stage (*p* = 0.046). However, the multivariate Cox's regression model included *TWIST1* expression levels, HR 1.99 (95% CI, 1.05–3.82) (*p* = 0.036) and gender, HR 2.07 (95% CI; 1.04–4.15) (*p* = 0.038) as independent prognosis factors for DFS, as well as the variable LNM, HR 3.4 (95% CI; 1.83–6.32) (Table 4).

**Table 4:** Unadjusted and adjusted analyses of the association between *TWIST* expression and disease-free survival of colon cancer patients.

Variable	Category	Unadjusted analysis			Adjusted analysis		
		HR	(95% CI)	*p* Value	HR	(95% CI)	*p* Value
Age at diagnosis	<71 vs >71	0.56	0.3–1.07	0.057	1.68	0.88–3.19	0.112
Sex of patients	Male vs female	1.895	0.96–3.76	0.067	2.07	1.04–4.15	0.038
Lymph node metastases	Yes vs No	3.57	1.94–6.57	<0.001	3.4	1.83–6.32	<0.001
Vascular invasion	Yes vs No	1.69	0.93–3.09	0.087			
Stage	II vs I	0.94	0.27–3.25	0.9			
	III vs I	3.39	1.02–11.28	0.046			
Histological grade	2 vs 1	1.91	0.93–3.93	0.08			
	3 vs 1	0.65	0.2–2.07	0.46			
Tumor side	Rectum vs colon	0.84	0.45–1.6	0.6			
*TWIST* expression	High vs low	1.54	0.83–2.84	0.17	1.99	1.05–3.82	0.036
Treatment Protocols	CT *vs* surg	4.52	2.16–9.43	<0.001			
	RT *vs* surg	1.44	0.51–4.03	0.49			
	CT + RT *vs* surg	3.15	1.35–7.38	0.008			

The blank cells correspond to variables that showed no independent relationship with OS in the adjusted analysis. CT, chemotherapy; RT, radiotherapy.

### *TWIST1* expression in patients with stages II and III tumors and Overall Survival

**Figure 4:**
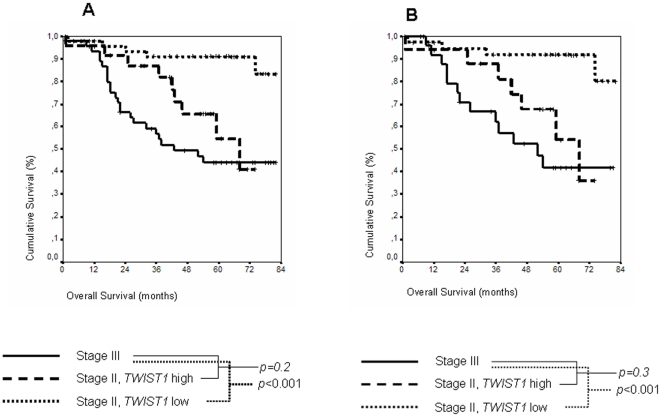
Kaplan-Maier OS curves in stages II and III. Kaplan-Meier curves and *p* values for OS at tumor stage III, tumor stage II with low *TWIST1* expression levels, and tumor stage II with high *TWIST1* expression levels. There is no significant difference between stage III and stage II with high *TWIST1* expression levels. A, global series. B, considering only colon cancer cases.

We compared the survival curves between patients with stage III tumors and patients with stage II tumors, dividing this group in two categories: those with low *TWIST1* expression levels and those with high expression levels (Fig. 4A). Unlike patients with stage III tumors, none of these patients received adjuvant chemotherapy. In this analysis, we did not observe difference at five-year OS between patients at stage III, 44.04% (95% CI; 28.98%–59.09%), and patients at stage II with high *TWIST1* expression levels, 54.53% (95% CI, 28.29%–80.77%). However, the difference was clear when patients in stage II expressing low *TWIST1* levels were compared with stage III patients (*p*<0.001). Moreover, by the end of the study (82 months) cumulative survival was higher in patients with stage III tumors, 44.04% (95% CI; 28.98%–59.09%), than in patients with stage II tumors and high *TWIST1* expression levels, 40.9% (95% CI; 10.52%–71.28%). This analysis was repeated, taking into account only colon cancer cases, not rectal cancer cases, in order to achieve greater homogeneity for the study. Again, the behavior of the different groups was very similar (Fig. 4B), confirming that this analysis was not affected by tumor location or treatment protocols, since all of the patients with stage III tumors were treated with chemotherapy before surgery.

## Discussion

We examined the expression of *TWIST1* in the normal and tumor tissues of a large series of 151 colorectal cancer patients. *TWIST1* expression was restricted to tumor tissues, indicating that it could be a tumor marker. Moreover, its expression was associated with certain pathological parameters linked to poor prognosis, such as LNM, which corroborates a publication in this field [22].

In our view, the most significant result was that *TWIST1* expression levels gave an independent prognostic factor, for both OS and DFS. Indeed, the detailed analysis of the correlation between *TWIST1* expression levels and OS and DFS, at each tumor pathological stage, showed an interaction between these two variables, pathological tumor stages and *TWIST1* expression. Therefore, for OS, *TWIST1* was a prognostic factor only at stages I and II, losing its prognostic value in advanced stages (III and IV). In a similar way, the study showed *TWIST1* as a prognostic factor for DFS only in stage I, losing this correlation in stages II and III.

The results found in our study have not been described in the literature on *TWIST1* expression and patients' prognosis. Although negative results have been reported between *TWIST1* expression and patient survival [28] in colon cancer cases, there are several publications that support this relationship, when analyzing the evolution of patients and mRNA *TWIST1* levels in colon cancer [29] and cervix carcinoma [30]. The expression of *TWIST1* and other functionally related genes, such as E-Cad, *SNAIL, SLUG* and *HIF-1α*, has also been studied in relation to survival in several tumor types, such as esophageal cancer [31], head and neck [32], [33] and bladder [34] carcinoma, with these relationships increasing when any of these genes are also overexpressed, as well as *TWIST1*. It is possible that a stage-by-stage analysis in some of these series would also provide results similar to ours. It would be reasonable to think that, if colon cancer develops by following steps [35], and tumor progression in these steps is caused by the accumulation of mutations in oncogenes and tumor suppressor genes, the accumulation of these at more advanced stages of disease could mimic the effects of overexpression of *TWIST1* on patients' survival.

Currently, the use of adjuvant chemotherapy for all patients with stage III colon cancer after resection is part of standard treatment around the world. However, the controversy about adjuvant treatment in stage II CRC is currently unresolved. The IMPACT B2 study pooled results from five trials in Dukes B2 colon cancer patients. This study did not show any benefit in five-year overall survival: results were 80% in the control group and 82% in the 5-fluorouracil and leucovorin group [36]. Nevertheless, the four NSABP adjuvant studies showed decreased death risk with adjuvant treatment, similar to the benefit obtained in stage C [37]. Another Dutch study reported a beneficial effect of 5-fluorouracil and levamisol adjuvant therapy in stage II patients, similar to the expected benefit in stage III ones [38]. Anyway, despite the lack of data, there is growing acceptance of an informal classification system, stratifying stage II patients by risk on the basis of clinical data, as a guide for deciding whether to use adjuvant therapy. Therefore, in stage II patients with high clinico-pathological risk (intestinal obstruction, perforation, tumor adherence, poor differentiation, vascular or lymphatic invasion), adjuvant therapy can reasonably be offered.

The analysis performed to study the behavior of *TWIST1* overexpression in relation to OS in subgroups of patients who received similar treatment protocols showed no significant differences, except for patients who had received no adjuvant therapy and thus patients in early stages. This supports the results found in the analysis of the complete series by stages, which showed that *TWIST1* is a discriminating factor in early stages of the disease.

Although, at stage III, *TWIST* expression did not differ in terms of OS and DFS, it could be relevant that the OS of stage-II patients with high *TWIST* expression was similar to that observed in patients at stage III. Moreover, the cumulative OS at the end of the study for this group of patients was higher than the cumulative OS in patients at stage II and high expression levels of *TWIST1*. These preliminary observations could support the idea of *TWIST1* expression as a tumor indicator at stage II, which could help select patients at greater risk who might benefit from adjuvant chemotherapy.

There is no biological explanation that justifies patients with stage I tumors and high *TWIST1* expression levels showing shorter cumulative survival, 33.3% (95% CI, 0%–86.65%), than the same category with stage II tumors, 54.53% (95% CI, 28.29%–80.77%). These results obtained from Kaplan-Meier analyses at each tumor stage may seem an artefact, due to the low number of samples in a group, such as stage I. However, our conclusions are supported by the results obtained from another approach, i.e. LNM status or treatment protocols in OS, where the study was not affected by the number of samples and showed that in both cases *TWIST1* mRNA expression levels have prognosis value only in early stages.

Cancer cells that are undergoing Epithelial-Mesenchymal Transition usually show deregulation of various genes. For instance, up-regulation of SNAIL1, ZEB1, ZEB2 or E12 in epithelial cells represses E-cadherin expression and induces EMT in several carcinomas [39]–[46]. Recently, the transcription factor TWIST1 has been added to the list of proteins that trigger EMT [4]. Yang et al. suggested that the expression of TWIST1 is essential in the intravasation step during the metastatic process. Expression of *TWIST1* by tumor cells might enhance the intravasation steps of metastasis. In this case, tumors expressing TWIST1 would display more aggressive behavior and trigger the intravasation steps, even though no visible metastases are observed.

We show that *TWIST1* expression levels may be an independent prognostic factor in patients with CRC. It may be well used in stage II to identify sub-groups of patients at high risk with a poor prognosis who might benefit from adjuvant chemotherapy.
